# Identification and Characterization of Four c-di-GMP-Metabolizing Enzymes from *Streptomyces ghanaensis* ATCC14672 Involved in the Regulation of Morphogenesis and Moenomycin A Biosynthesis

**DOI:** 10.3390/microorganisms9020284

**Published:** 2021-01-30

**Authors:** Desirèe Nuzzo, Roman Makitrynskyy, Olga Tsypik, Andreas Bechthold

**Affiliations:** Department of Pharmaceutical Biology and Biotechnology, Institute of Pharmaceutical Sciences, Albert-Ludwigs University, 79104 Freiburg, Germany; desiree.nuzzo@pharmazie.uni-freiburg.de (D.N.); olga.tsypik@pharmazie.uni-freiburg.de (O.T.)

**Keywords:** *Streptomyces ghanaensis* ATCC14672, moenomycin A, c-di-GMP, diguanylate cyclases, phosphodiesterases, morphogenesis, antibiotic, natural products, regulation

## Abstract

Diguanylate cyclases (DGCs) and phosphodiesterases (PDEs) are essential enzymes deputed to maintain the intracellular homeostasis of the second messenger cyclic dimeric (3′→5′) GMP (c-di-GMP). Recently, c-di-GMP has emerged as a crucial molecule for the streptomycetes life cycle, governing both morphogenesis and secondary metabolite production. Indeed, in *Streptomyces ghanaensis* ATCC14672 c-di-GMP was shown to be involved in the regulatory cascade of the peptidoglycan glycosytransferases inhibitor moenomycin A (MmA) biosynthesis. Here, we report the role of four c-di-GMP-metabolizing enzymes on MmA biosynthesis as well as morphological progression in *S. ghanaensis*. Functional characterization revealed that RmdA_gh_ and CdgA_gh_ are two active PDEs, while CdgE_gh_ is a DGC. In vivo, overexpression of *rmdA_gh_* and *cdgA_gh_* led to precocious sporulation, whereas overexpression of *cdgE_gh_* and *cdgD_gh_* (encoding a predicted DGC) caused an arrest of morphological development. Furthermore, we demonstrated that individual deletion of *rmdA_gh_*, *cdgA_gh_,* and *cdgD_gh_* enhances MmA accumulation, whereas deletion of *cdgE_gh_* has no impact on antibiotic production. Conversely, an individual deletion of each studied gene does not affect morphogenesis. Altogether, our results show that manipulation of c-di-GMP-metabolizing enzymes represent a useful approach to improving MmA production titers in *S. ghanaensis*.

## 1. Introduction

Streptomycetes are filamentous soil bacteria with a complex, strictly-regulated morphological development. Their life cycle begins with the germination of spores to vegetative mycelium. In response to stress signals, aerial hyphae are formed, which ultimately differentiate into spore chains [1,2]. Due to their ability to produce plenty of various secondary metabolites, streptomycetes are the most exploited *Actinobacteria* to combat multidrug-resistant (MDR) pathogens.

Moenomycin A (MmA) is the founding member of a class of secondary metabolites known as moenomycins. The latter consist of a mixture of phosphoglycolipids produced by several *Streptomyces* strains including *Streptomyces ghanaensis* ATCC14672 [3]. The great interest in this family of antibiotics arises from their high potency and unique mechanism of action. Indeed, moenomycins are the only known natural inhibitors of peptidoglycan 

glycosyltransferases, enzymes involved in the penultimate step of bacterial cell-wall biosynthesis [4]. Moreover, they show a broad spectrum of action against pathogenic bacteria—including vancomycin-resistant enterococci (VRE) and methicillin-resistant *Staphylococcus aureus* (MRSA)—making them a promising lead against MDR microorganisms [4]. Nevertheless, wild-type *S. ghanaensis* produces a low amount of MmA, therefore many studies on the regulation of its biosynthesis have been addressed to generate overproducer strains.

Typically, secondary metabolite biosynthesis is fine-tuned regulated by two interconnected networks, comprising global regulators (e.g., Bld and Whi) [1] and cluster-situated regulators (CSRs) [5]. Surprisingly, no CSRs were identified within MmA biosynthetic gene clusters (known as *moe* clusters) [3], suggesting that its biosynthesis is differently governed. Indeed, it was shown that the pleiotropic regulators WblA [6], AdpA, BldA, and AbsB [7] control MmA production. WblA and AdpA are wide-distributed transcriptional regulators in *Streptomyces* species, governing both morphological development and antibiotic biosynthesis [8,9,10,11,12]. BldA is a tRNA^Leu^_UAA_, the only tRNA able to translate the rarest UUA codon in streptomycetes [13].

Intriguingly, expression of *wblA*, *adpA*, and *bldA* is controlled by the master pleiotropic regulator BldD [14,15]. Acting as a transcriptional regulator of morphogenesis and antibiotic production [14,16], BldD governs the expression of dozens of genes in streptomycetes [14,17].

In 2014, Tschowri et al. showed that the activity of BldD is regulated by the second messenger cyclic dimeric (3′→5′) GMP (c-di-GMP). In fact, BldD acts as homodimer and its dimerization is mediated by a tetrameric form of c-di-GMP [17]. Additionally, a recent study revealed another c-di-GMP-sensing effector in streptomycetes. Indeed, in *S. venezuelae* the binding between the sporulation-specific σ^WhiG^ factor and its anti-σ is bridged by a c-di-GMP dimer, leading to the block of sporogenesis [18]. Consequently, alterations of intracellular c-di-GMP levels severely affect the streptomycetes life cycle.

In order to preserve its homeostasis, the turnover of the cyclic dinucleotide is strictly maintained by the antagonistic activities of two classes of enzymes: the diguanylate cyclases (DGCs) and the phosphodiesterases (PDEs). DGC enzymes synthesize c-di-GMP using two molecules of GTP. Their active site consists of the highly-conserved GGDEF (Gly-Gly-(Asp/Glu)-Glu-Phe) domain [19,20]. PDEs are characterized by the catalytic EAL (Glu-Ala-Leu) domain, which is responsible for the degradation of c-di-GMP into the linear dinucleotide 5′-phosphoguanylyl-(3′→5′)-guanosine (pGpG) [21,22]. Moreover, PDEs containing the less frequent HD-GYP (His-Asp)-(Gly-Tyr-Pro) domain hydrolyze c-di-GMP into two GMP molecules [23]. Commonly, bacteria possess more than one DGC or PDE, therefore their activity is controlled on both genetic and post-translational levels. Indeed, many c-di-GMP-metabolizing enzymes are often associated with sensor domains (e.g., REC, PAS and/or GAF), which enable these proteins to respond to environmental stimuli [24].

In past years, many studies have highlighted the importance of DGCs and PDEs in *Streptomyces* species. [14,15,17,25,26,27,28,29]. In fact, manipulation of genes encoding for these enzymes resulted in remarkable effects on morphological progression and secondary metabolite production. For example, in *S. coelicolor* overexpression of the DGCs *cdgA*, *cdgB*, and *cdgD* caused the arrest of morphological development and reduced the production of the blue-pigmented antibiotic actinorhodin [14,25,26]. Furthermore, upon simultaneous deletion of *rmdA* and *rmdB* (encoding two PDEs), the resulting strain displayed a “bald” phenotype (e.g., absence of aerial mycelium) and increased concentration of c-di-GMP [27]. Analogously, in *S. venezuelae* the DGC CdgC and orthologs of CdgA, CdgB, RmdA, and RmdB are also involved in morphogenesis [28]. In fact, it was shown that in *cdgB*, *cdgC*, *rmdA*, and *rmdB* null-mutants the transcription of numbers of genes associated with chaplins and rodlins production, hydrophobic sheath formation, and cell division was strongly affected [29].

Recently, we demonstrated that orthologs of CdgB, RmdB, and CdgC in *S. ghanaensis* play a crucial role in both MmA biosynthesis and morphological development [15,30].

In this work, we studied the function of *cdgE_gh_* (*ssfg_02707*), *cdgD_gh_* (*ssfg_02343*), *cdgA_gh_* (*ssfg_04551*), and *rmdA_gh_* (*ssfg_00725*), the other four genes encoding putative c-di-GMP-metabolizing enzymes from *S. ghanaensis*. Coupling in vitro and in vivo experiments, we elucidated the activity of the corresponding enzymes and we revealed their importance in morphogenesis as well as MmA production. Particularly, we showed that CdgE_gh_ is a DGC, whereas RmdA_gh_ is a bifunctional enzyme able to both synthesize and degrade c-di-GMP. Intriguingly, we demonstrated that the putative DGC CdgA_gh_ actually functions solely as PDE in *S. ghanaensis*. In addition, overexpression of all genes strongly affected morphological development, whereas MmA accumulation was mostly altered in *rmdA_gh_*, *cdgA_gh_* and *cdgD_gh_* null-mutants as well as in the *S. ghanaensis* strain carrying a mutated allele of *rmdA_gh_*. Altogether, these results demonstrate that manipulation of c-di-GMP-metabolizing enzymes is a useful approach to increase antibiotic titer in *S. ghanaensis*.

## 2. Materials and Methods

### 2.1. Bioinformatic Analysis

SMART domain database (https://smart.embl.de) [31] was used to analyze CdgE_gh_, CdgD_gh_, RmdA_gh_, and CdgA_gh_ protein sequences. Multiple sequence alignments of PDE and DGC domains were performed using the Clustal Omega software (https://www.ebi.ac.uk/Tools/msa/) [32] and the figures were created using ESPript 3.0 software (http://espript.ibcp.fr) [33].

### 2.2. Strains, Plasmids, and Growth Conditions

All plasmids and bacteria strains used in this work are listed in Table S1. *Escherichia coli* strains were grown at 37 °C in Luria-Bertani (LB) medium. Soya mannitol agar (SFM), oatmeal agar, and tryptic soy broth (TSB) liquid media were used to grow *S. ghanaensis* strains at 37 °C. Where necessary, appropriate antibiotics were added to the media.

### 2.3. DNA Manipulation and Intergeneric Conjugation

All primers used in this work are listed in Table S2. Polymerase chain reactions (PCR) were carried out using Phusion polymerase (ThermoFisher, Waltham, MA, USA). Chromosomal and plasmid DNA were isolated following standard procedures [34]. Sequencing, PCR, or enzymatic digestion were utilized to verify the constructed plasmids. Intergeneric conjugation was applied to transfer plasmids from *E. coli* ET12567 (carrying the helper plasmid pUZ8002) to *S. ghanaensis*, as described previously [35].

### 2.4. Proteins Production and Purification

Full-length N-terminal His6-tagged proteins were produced as follows. DNA sequences encoding CdgE_gh_, RmdA_gh_ and CgdA_gh_ were amplified from the chromosomal DNA by PCR using primers 02707_exp_for/02707_exp_rev, rmdA_exp_for/rmdA_exp_rev, and cdgA_exp_for/cdgA_exp_rev, respectively. The resulting fragments *cdgE_gh_* and *cdgA_gh_* were digested with NdeI and XhoI and cloned into NdeI-XhoI linearized pET28a vector, yielding pETcdgE and pETcdgA, respectively. Instead, the PCR product corresponding to the *rmdA_gh_* coding sequence was digested with NdeI and HindIII and ligated into NdeI-HindIII linearized pET28a vector, yielding pETrmdA. To generate a mutated version of CdgA_gh_ (named CdgA_gh_^AAL^), PCR mutagenesis was used to amplify a 7,3 kb DNA fragment from pETcdgA using primers cdgA_AAL_for and cdgA_AAL_rev (carrying a E412A substitution). The resulting amplicon was first treated with T4 Polynucleotide kinase and then self-ligated, yielding pETcdgA^AAL^. The latter was used as a template to amplify the *cdgA_gh_^AAL^* allele (primers cdgA_BamHI_for and cdgA_exp_rev). The resulting PCR product was first digested with BamHI and XhoI and then ligated into the linearized BamHI-XhoI pET32a vector, yielding pET32acdgA^AAL^. The same approach was used to create a mutated version of CdgE_gh_ (named CdgE_gh_^AADEF^). A 6,4 kb DNA fragment from pETcdgE was amplified using primers (02707_AADEF_for and 02707_AADEF_rev) carrying the G271A and G272A substitutions. The final circularized product was named pETcdgE^AADEF^.

In order to produce a truncated N-terminal His6-tagged version of RmdA_gh_, the DNA sequence encoding solely for the GGDEF domain of RmdA_gh_ (named RmdA_gh_^GGDEF^) was amplified by PCR using primers rmdA_exp_for and rmdA_GGDEF_rev. The obtained fragment was digested with NdeI and HindIII and cloned into the NdeI-HindIII linearized pET28a vector, yielding pETrmdA^GGDEF^.

pETcdgE, pETcdgE^AADEF^, pETcdgA, and pETrmdA^GGDEF^ were individually introduced into *E. coli* BL21 Star (DE3), whereas pETrmdA and pET32acdgA^AAL^ were individually introduced into *E. coli* BL21 Star (DE3) pLysS. Strains were grown at 37 °C to OD_600_ of 0.5 and protein production was induced with 0.2 mM IPTG (for production of RmdA_gh_ 0.5 mM IPTG was used). Afterwards, cultures were incubated at 22 °C for 5 h and then harvested by centrifugation. The resulting pellet was resuspended in equilibration buffer (see Table S3) and cells were lysed by French-press. The lysate was centrifuged for 30 min at 4 °C and proteins were purified from a soluble fraction by affinity chromatography using Ni-NTA beads with an equilibration buffer containing 200 mM imidazole. For CdgE_gh_, CdgE_gh_^AADEF^, RmdA_gh_, CdgA_gh,_ and RmdA_gh_^GGDEF^ proteins, size-exclusion chromatography was performed using the ÄKTA fast protein liquid chromatography (FPLC) system, equipped with a Superdex 200 HR 16/60 column. Finally, proteins were stored at –80 °C in a storage buffer (see Table S3). For CdgA_gh_^AAL^, pure eluted fractions were pooled together and washed from imidazole through dialysis with a storage buffer containing 50 mM Tris-HCl, 0.25 M NaCl, 1 mM MgCl_2_, 1 mM dithiotreitol, and 5% glycerol, pH 7.5.

### 2.5. Diguanylate Cyclase and Phosphodiesterase Enzymatic In Vitro Assays

Enzymatic in vitro assays were performed and analyzed as described previously [15,30]. To test a DGC activity, 5 µM purified His6-CdgE_gh_, His6-RmdA_gh_^GGDEF^, His6-RmdA_gh_, and His6-CdgA_gh_ in cyclase buffer [50 mM Tris-HCl (pH 8.0), 50 mM NaCl, 10 mM MgCl_2_] were individually incubated with 200 µM GTP. To test a PDE activity, 5 µM purified His6-RmdA_gh_, His6-CdgA_gh_, and Trx-His6-CdgA_gh_^AAL^ were individually added to a reaction mixture containing 200 µM c-di-GMP, 50 mM Tris-HCl [pH 8.0], 50 mM NaCl, and 10 mM MgCl_2_. Following 2 h incubation at 37 °C, reactions were stopped first by adding 10 mM CaCl_2_ and then by heating at 70 °C for 10 min. Reaction products were analyzed by LC–MS. Standards were prepared by using purchased c-di-GMP (InvivoGen, San Diego, California, USA) and GTP (Sigma-Aldrich, St. Louis, MI, USA).

### 2.6. Gene Overexpression Experiments

In order to perform gene-overexpression experiments, the coding sequence of each target gene (*cdgE_gh_*, *cdgD_gh_*, *rmdA_gh_* and *cdgA_gh_*) was amplified by PCR, using primers 02707_exp_for/02707_exp_rev, 02343_exp_for/02343_exp_rev, rmdA_exp_for/rmdA_exp_rev, and cdgA_exp_for/cdgA_exp_rev, respectively. The PCR products corresponding to *cdgE_gh_*, *cdgD_gh_*, and *cdgA_gh_* were digested with NdeI and XhoI and cloned under the control of the constitutive promoter *ermEp* into the NdeI-XhoI digested integrative pIJ10257 vector. Instead, the fragment containing the coding sequence of *rmdA_gh_* was digested with NdeI and HindIII and ligated into the pIJ10257 vector, linearized by NdeI and HindIII. The resulting plasmids pIJcdgE, pIJcdgD, pIJcdgA, and pIJrmdA were individually transferred into *S. ghanaensis* by conjugation, leading to the formation of four different strains (*S. ghanaensis* + pIJcdgE, *S. ghanaensis* + pIJcdgD, *S. ghanaensis* + pIJcdgA, and *S. ghanaensis* + pIJrmdA).

Next, overexpression of *cdgA_gh_* and *rmdA_gh_* alleles carrying a point mutation in their EAL motif (Glu to Ala) was performed as following. The DNA fragment containing *cdgA_gh_^AAL^* was digested from pETcdgA^AAL^ plasmid using NdeI and XhoI and subcloned into the NdeI-XhoI linearized pIJ10257 vector, yielding pIJcdgA^AAL^.

To generate a mutated version of *rmdA_gh_* (named *rmdA_gh_^AAL^*), PCR mutagenesis was used to amplify a 7.4 kb DNA fragment from pETrmdA using primers designed to bring the point substitution E485A (rmdA_AAL_for and rmdA_AAL_rev). The obtained amplicon was treated with T4 Polynucleotide kinase and then self-ligated, yielding pETrmdA^AAL^. The latter was digested with NdeI and HindIII and the resulting *rmdA_gh_^AAL^* fragment was subcloned into the NdeI-HindIII linearized pIJ10257 vector, yielding pIJrmdA^AAL^. Finally, pIJcdgA^AAL^ and pIJrmdA^AAL^ were transferred into the *S. ghanaensis* strain by intergeneric conjugation.

### 2.7. Gene Deletion and Complementation Experiments

*S. ghanaensis* Δ*cdgE_gh_*, *S. ghanaensis* Δ*cdgD_gh_*, *S. ghanaensis* Δ*rmdA_gh_*, and *S. ghanaensis* Δ*cdgA_gh_* were generated through homologous recombination, as described previously [30]. Briefly, the DNA fragments corresponding to *cdgE_gh_*, *cdgD_gh_*, *rmdA_gh_*, and *cdgA_gh_* coding sequences along with their flanking regions were amplified by PCR (primers: 02707_del_for/02707_del_rev; 02343_del_for/02343_del_rev; rmdA_del_for/rmdA_del_rev; cdgA_del_for/cdgA_del_rev). Then, the obtained amplicons were ligated into the EcoRV-digested pBluescriptKS+ vector, yielding pBluecdgE, pBluecdgD, pBluermdA, and pBluecdgA, respectively. Next, each plasmid was individually introduced into *E. coli* BW25113/pIJ790, and REDIRECT technology [36] was used to replace each target gene with the apramycin resistance cassette [*aac(3)IV*], flanked by *loxP* sites. The resulting fragments cdgE::aac(3)IV, cdgD::aac(3)IV, rmdA::aac(3)IV, and cdgA::aac(3)IV were amplified by PCR and individually cloned into the suicide pKGLP2 vector, linearized with EcoRV. The obtained pKGcdgE::aac(3)IV, pKGcdgD::aac(3)IV, pKGrmdA::aac(3)IV and pKGcdgA::aac(3)IV were individually transferred into *S. ghanaensis* by conjugation, and double-crossover mutants were selected for hygromycin sensitivity and apramycin resistance. Finally, marker-free mutants were generated by introducing the Cre-expressing helper plasmid pUWLCre into mutants by conjugation [37]. The deletion of the gene marker from the genome of each mutant was confirmed by PCR.

For complementation of *S. ghanaensis* Δ*cdgD_gh_* and *S. ghanaensis* Δ*rmdA_gh_*, plasmids pIJcdgD and pIJrmdA were used, respectively. To complement the *S. ghanaensis* Δ*cdgA_gh_* mutant, the *cdgA_gh_* coding sequence, along with its own promoter region, was amplified by PCR (primers cdgA_compl_for and cdgA_compl_rev) and digested with XbaI and EcoRI. The fragment was cloned into the XbaI-EcoRI-digested φC31-based integrative vector pSET152, to gain pSETcdgA. The latter was then transferred into *S. ghanaensis* Δ*cdgA_gh_* by conjugation.

### 2.8. Quantitative Analysis of MmA Accumulation

Moenomycin production was quantified as described elsewhere [38,39]. Briefly, *S. ghanaensis* strains were grown four days in 50 mL TSB, in triplicate. Afterwards, the culture was centrifuged and the pellet was resuspended in 10 mL methanol under shaking overnight. The resulting extracts were concentrated *in vacuo*, dissolved in methanol and analyzed by HPLC–MS, as described previously [15]. The experiments were repeated at least three times and the levels of antibiotic were referred back to the equal amount of dry biomass (10 mg) in different strains. In this work, moenomycin corresponds to the mixture of the two main compounds MmA ([M-H]^−^ = 1580.6 Da) and the precursor nosokomycin B (NoB; [M-H]^−^ = 1484.6 Da). The mean value of moenomycin mass peak area of *S. ghanaensis* wild-type was taken as 100% and the data shown in Figure 4 represent the mean value of independent experiments. Error bars indicate the standard deviations.

## 3. Results

### 3.1. Bioinformatic Analysis of CdgE_gh_, CdgD_gh_, RmdA_gh_ and CdgA_gh_, Four Putative c-di-GMP-Metabolizing Enzymes from S. ghanaensis

c-di-GMP-metabolizing enzymes are highly-conserved and widely spread among *Streptomyces* species. Interestingly, streptomycetes possess multiple genes encoding for DGC/PDE enzymes. Similarly, *in silico* analysis of *S. ghanaensis* genome revealed the presence of nine genes predicted to encode DGCs and PDEs (Table 1). Upon previous characterization of *cdgB_gh_*, *rmdB_gh_*, and *cdgC_gh_* (*ssfg_02181*) [15,30], we focused our attention on *cdgE_gh_*, *cdgD_gh_*, *rmdA_gh_*, and *cdgA_gh_*. CdgE_gh_ (382-aa) and CdgD_gh_ (204-aa) are two putative DGCs consisting of a GAF-GGDEF and a GGDEF domain architecture, respectively. The 649-aa CdgA_gh_ and the 714-aa RmdA_gh_ proteins both contain highly-conserved GGDEF and EAL domains, preceded by an N-terminal PAS and PAS-PAC domain, respectively (Figure S1).

In order to predict the putative function of these enzymes, multiple sequence alignment was performed using Clustal Omega software. As a reference, the well-characterized GGDEF domain of PleD (a DGC from *Caulobacter crescentus*) [19] and EAL domain of RocR (a PDE from *Pseudomonas aeruginosa*) [40] were chosen.

**Figure 1:**
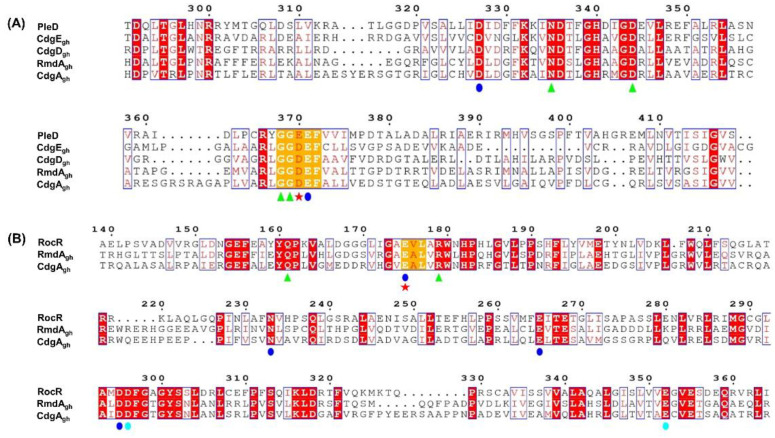
Protein sequence alignments of CdgE_gh_, CdgD_gh_, RmdA_gh_, and CdgA_gh_. (**A**) Sequence alignment of CdgE_gh_, CdgD_gh_, RmdA_gh_, and CdgA_gh_ GGDEF domains with the active GGDEF domain of PleD from *Caulobacter crescentus*. The residues are numbered according to the sequence of PleD. (**B**) Sequence alignment of RmdA_gh_ and CdgA_gh_ EAL domains with the active EAL domain of RocR from *Pseudomonas aeruginosa*. The residues are numbered according to the sequence of RocR. Conservative amino acids involved in substrate binding (green triangles), metal coordination (blue and cyan circles), and catalysis (red star) are shown. Alignments were performed using Clustal-Omega (https://www.ebi.ac.uk/Tools/msa/) and the figures were generated using ESPript 3.0 (http://espript.ibcp.fr).

As shown in Figure 1A, all four enzymes possess in their GGDEF domain the amino acids crucial for DGC activity. Particularly, each protein sequence carries the canonical DxLT motif, which is required for the formation of a stabilizing “wide-turn” structure and is located at the beginning of the GG(D/E)EF domain [41]. Moreover, residues involved in metal ion coordination (D327 and E371) [19], GTP binding (N335, D344, G368, and G369) [24,41] and catalysis (E/D370) [42,43] were found to be well-conserved, suggesting that all these enzymes could function as DGCs. Interestingly, in comparison to PleD, CdgE_gh_, CdgD_gh_, RmdA_gh_, and CdgA_gh_ sequences all lack the canonical RxxD inhibition site, involved in the negative feedback regulation of many DGC enzymes.

Sequence alignment of RmdA_gh_ and CdgA_gh_ EAL domains compared to RocR showed that both proteins could also function as PDEs. In fact, critical residues essential for catalysis (E175) [40,43,44], substrate binding (Q161 and R179) [40], and metal coordination (E175, N233, E265, D295, D296, and E352) [40,45] are all present and identical among the sequences (Figure 1B). This evidence indicates that CdgA_gh_ as well as RmdA_gh_ possess putative enzymatically active GGDEF and EAL domains, therefore they likely act as bifunctional enzymes.

### 3.2. In Vitro Determination of RmdA_gh_, CdgA_gh_ and CdgE_gh_ Enzymatic Activities

Orthologs of CdgE_gh_, CdgD_gh_, RmdA_gh_, and CdgA_gh_ were previously found in the model organisms *S. coelicolor* and *S. venezuelae*. In *S. coelicolor*, CdgA was described as a DGC, but its catalytic activity has not yet been proved *in vitro [14]*. On the contrary, an ortholog of CdgD_gh_ in *S. coelicolor* (CdgD) was confirmed to work as a DGC [26]. RmdA was found to function solely as PDE in *S. coelicolor* [27] and to act as bifunctional enzyme in *S. venezuelae [29]*. Finally, CdgE (ortholog of CdgE_gh_) was recently proved to be an active DGC in *S. venezuelae [29]*. Similarly, our *in silico* analysis suggested that CdgE_gh_ and CdgD_gh_ are likely two DGCs, whereas RmdA_gh_ and CdgA_gh_ could possess both c-di-GMP synthesis and hydrolytic activities.

In order to prove an enzymatic activity in vitro, RmdA_gh_, CdgA_gh_, and CdgE_gh_ were heterologously produced in *E. coli*. Despite all our attempts, we were unable to generate an *E. coli* strain producing soluble CdgD_gh_.

Full-length RmdA_gh_, CdgA_gh_, and CdgE_gh_ proteins were fused with an N-terminal His6-tag and purified to homogeneity. To test a PDE activity, purified proteins were individually added to a reaction mixture containing 200 µM c-di-GMP. Conversely, 200 µM GTP were used as a substrate for a DGC activity assay, at the presence of either RmdA_gh_, CdgA_gh_, or CdgE_gh_. As a control, a mixture without enzyme was used. After incubation for two hours at 37 °C, the reaction products were analyzed by LC–MS.

**Figure 2:**
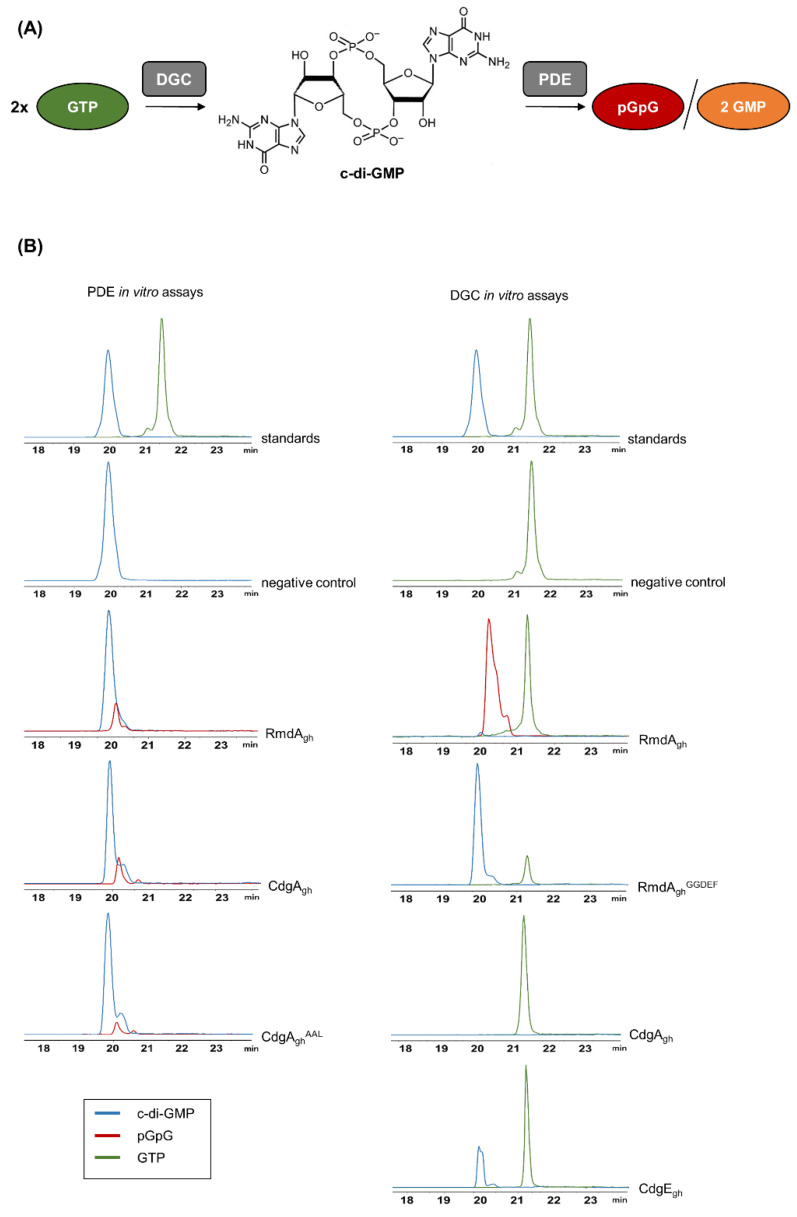
Schematic diagram of cyclic dimeric (3′→5′) GMP (c-di-GMP) synthesis and degradation and LC–MS chromatograms of in vitro phosphodiesterase (PDE) and diguanylate cyclase (DGC) assays. (**A**) c-di-GMP is synthesized by the DGC enzyme, starting from two molecules of GTP. Instead, PDE enzymes hydrolyze c-di-GMP to linear pGpG or to two molecules of GMP. (**B**) Upon PDE in vitro assays, the EIC corresponding to the expected [M-H]^−^ ion of pGpG (*m/z* = 707.1) was detected in both reaction mixtures containing RmdA_gh_ and CdgA_gh_, respectively, but not in the negative control (without enzyme). Mutation in the conservative EAL domain of CdgA_gh_ (CdgA_gh_^AAL^) strongly reduced the hydrolytic activity. Upon DGC in vitro assays, the EIC corresponding to the expected [M-H]^-^ ion of c-di-GMP (*m/z* = 689.1) was detected in the reaction mixtures containing a truncated version of RmdA_gh_ (RmdA_gh_^GGDEF^), carrying solely its GGDEF domain, and CdgE_gh_, respectively. No conversion of GTP to c-di-GMP was observed in the reaction mixture containing CdgA_gh_, whereas the predominant reaction product detected in the mixture containing RmdA_gh_ was pGpG.

As shown in Figure 2, RmdA_gh_ was able to hydrolyze c-di-GMP to linear pGpG (*m/z* = 707.1 [M-H]^−^; see also MS spectra in Figure S2). Intriguingly, the predominant product observed in the reaction mixture containing RmdA_gh_ and GTP was also pGpG, demonstrating that RmdA_gh_ is indeed a bifunctional enzyme able to synthesize and then degrade c-di-GMP. To confirm that RmdA_gh_ can produce c-di-GMP, we generated a truncated version of the enzyme (named RmdA_gh_^GGDEF^) consisting solely of its GGDEF domain. After two hours of incubation, the expected molecular ion of c-di-GMP (*m/z* = 689.1 [M-H]^−^) was indeed observed in RmdA_gh_^GGDEF^ (Figure 2).

To examine CdgA_gh_ for a dual activity, we incubated the enzyme into two different reaction mixtures, one containing 200 µM GTP and the other 200 µM c-di-GMP. Surprisingly, no cyclization was observed (Figure 2) but solely the degradation of c-di-GMP into pGpG. To prove that this PDE activity derived from CdgA_gh_, we introduced a point mutation (E412A) in its EAL motif, generating CdgA_gh_^AAL^ protein. Upon mutagenesis, CdgA_gh_^AAL^ resulted poorly soluble. Hence, to improve its solubility, the protein was fused N-terminally with a Trx-His6tag. The resulting Trx-His6-tagged CdgA_gh_^AAL^ was purified and used for an in vitro assay. As a result, the hydrolytic activity was reduced of 1.8-fold in comparison to the wild-type protein, indicating that mutations in the EAL domain of CgdA_gh_ strongly affect its catalytic activity. Moreover, we found that the mutation did not trigger the activity of the GGDEF domain, since no c-di-GMP accumulation was observed upon incubation of CdgA_gh_^AAL^ with GTP (Figure S3).

Finally, we tested CdgE_gh_ for a DGC activity. As anticipated from *in silico* analysis, CdgE_gh_ was able to convert GTP into c-di-GMP (Figure 2). To validate its cyclase activity, a mutated version of His6-tagged-CdgE_gh_ (named CdgE_gh_^AADEF^) carrying both G271A and G272A substitutions in its GGDEF domain was purified. Upon reaction, no c-di-GMP was synthesized (Figure S3), showing that a conservative GGDEF motif is crucial for the catalysis.

In summary, these results demonstrated that in *S. ghanaensis* RmdA_gh_ is a bifunctional enzyme and CdgE_gh_ is a DGC. Additionally, we showed for the first time an in vitro activity for CdgA_gh_, proving that this enzyme acts solely as PDE in *S. ghanaensis.*

### 3.3. Overexpression of cdgE_gh_, cdgD_gh_, rmdA_gh_ and cdgA_gh_ Remarkably Alters Morphogenesis in S. ghanaensis

In past years, manipulation of c-di-GMP-metabolizing enzymes was shown to severely alter morphological progression in *Streptomyces* species. [14,15,25,26,27,28,29,30]. Hence, we aimed to study the influence of CdgE_gh_, CdgD_gh_, RmdA_gh_, and CdgA_gh_ on morphogenesis in *S. ghanaensis*.

For this purpose, we first constructed four different marker-free null-mutants through homologous recombination, leading to *S. ghanaensis* Δ*cdgE_gh_*, *S. ghanaensis* Δ*cdgD_gh_*, *S. ghanaensis* Δ*rmdA_gh_*, and *S. ghanaensis* Δ*cdgA_gh_*. Mutant strains were cultivated on SFM agar and oatmeal agar for four days at 37 °C. Surprisingly, no significant differences were observed in comparison to the wild-type strain (Figure S4).

**Figure 3:**
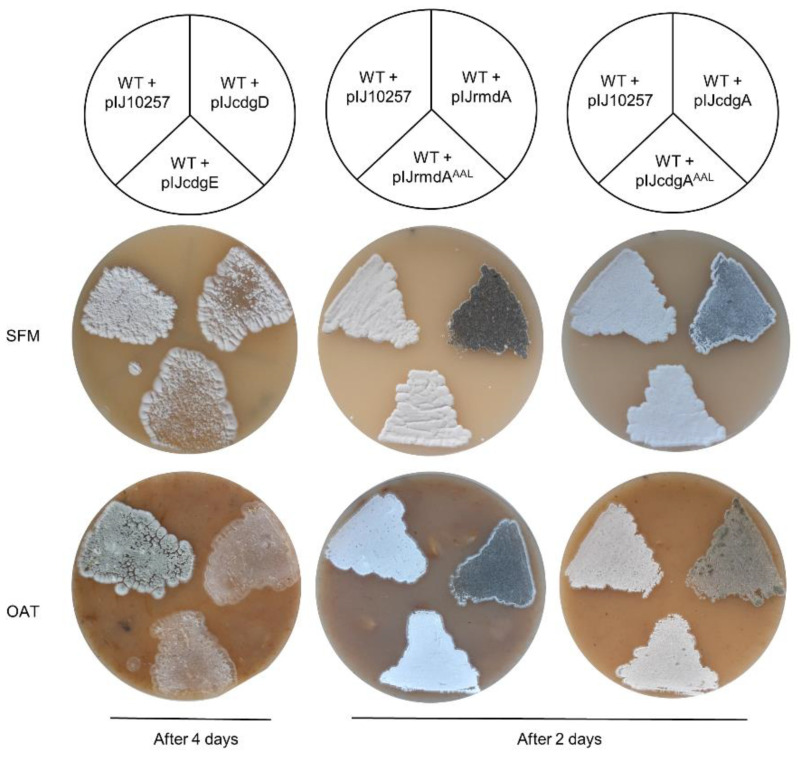
Individual overexpression of *cdgE_gh_, cdgD_gh,_ rmdA_gh_*, and *cdgA_gh_*, severely affects morphogenesis of *S. ghanaensis* (wild type, WT). After four days of growth, *cdgE_gh_*-overexpressed *S. ghanaensis* strain (WT + pIJcdgE) and *cdgD_gh_*-overexpressed *S. ghanaensis* strain (WT + pIJcdgD) were arrested in their morphological progression and barely developed aerial mycelium, in comparison to the control strain (WT + pIJ10257), carrying the pIJ10257 empty vector. Conversely, after two days of growth, *rmdA_gh_*-overexpressed *S. ghanaensis* strain (WT + pIJrmdA) and *cdgA_gh_*-overexpressed *S. ghanaensis* strain (WT + pIJcdgA) displayed accelerated sporulation. Overexpression of *rmdA_gh_* and *cdgA_gh_* carrying a point mutation in their EAL domain (WT + pIJrmdA^AAL^ and WT + pIJcdgA^AAL^, respectively) reversed the precocious sporogenesis. All strains were grown on SFM agar and oatmeal (OAT) agar at 37 °C.

In contrast to gene deletion experiments, gene overexpression led to remarkable consequences on morphological development. First, we used the φBT1-based integrative vector pIJ10257 to generate four plasmids (pIJcdgE, pIJcdgD, pIJrmdA, and pIJcdgA), where the corresponding gene was placed under control of the strong constitutive promoter *ermEp*. Next, the resulting plasmids were individually transferred into *S. ghanaensis* and analysis on morphogenesis was performed. As shown in Figure 3, overexpression of *cdgE_gh_* and *cdgD_gh_* severely affected morphological progression. Indeed, after four days of growth on SFM agar both *S. ghanaensis* + pIJcdgE and *S. ghanaensis* + pIJcdgD strains were strongly delayed in the development of aerial mycelium, in comparison to the control strain carrying an empty vector. Moreover, the most drastic difference was observed on oatmeal agar, where both mutants were unable to generate a typical aerial mycelium and remained “bald”, whereas the control strain developed mature spores. Conversely, *S. ghanaensis* + pIJrmdA and *S. ghanaensis* + pIJcdgA quickly passed the aerial mycelium phase and sporulated already after two days of growth on SFM and oatmeal agar, whereas the control strain still remained white (Figure 3). To prove that the obtained phenotype was caused by a PDE activity, we replaced the glutamate with alanine in the EAL motif of *rmdA_gh_* as well as *cdgA_gh_*. The resulting genes (*rmdA_gh_^AAL^* and *cdgA_gh_^AAL^*) were used to construct the plasmids pIJrmdA^AAL^ and pIJcdgA^AAL^. As depicted in Figure 3, mutation in the EAL domain of both genes completely reversed the precocious sporulation, confirming that the EAL motif is crucial for the activity in both *rmdA_gh_* and *cdgA_gh_* observed phenotypes.

Altogether, these results showed that overexpression of *cdgE_gh_*, *cdgD_gh_*, *rmdA_gh_*, and *cdgA_gh_* severely alters morphological development in *S. ghanaensis*. Particularly, we demonstrated that CdgE_gh_ and CdgD_gh_ act as DGCs in vivo, leading to the arrest of morphological progression. On the contrary, RmdA_gh_ and CdgA_gh_ function as PDEs and their overexpression strongly induces sporogenesis.

### 3.4. Genetic Manipulation of c-di-GMP-Metabolizing Enzymes Leads to Variations in MmA Accumulation

Recently, we showed that the PDE RmdB_gh_ and the two DGCs CdgB_gh_ and CdgC_gh_ are strongly involved in the regulation of MmA production in *S. ghanaensis* [15,30]. In order to investigate whether *cdgE_gh_*, *cdgD_gh_*, *rmdA_gh_*, and *cdgA_gh_* may also have a role in antibiotic biosynthesis, we analyzed MmA accumulation in both null-mutants and gene-overexpressed strains.

**Figure 4:**
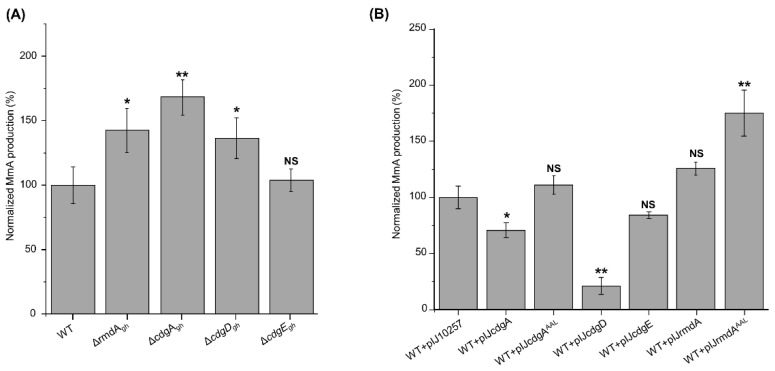
Levels of moenomycin production accumulated in the biomass of different *S. ghanaensis* strains. (**A**) *S. ghanaensis* wild-type (WT), *rmdA_gh_* null-mutant (Δ*rmdA_gh_*), *cdgA_gh_* null-mutant (Δ*cdgA_gh_*), *cdgD_gh_* null-mutant (Δ*cdgD_gh_*) and *cdgE_gh_* null-mutant (Δ*cdgE_gh_*). (**B**) *S. ghanaensis* wild-type carrying empty pIJ10257 vector (WT + pIJ10257), *cdgA_gh_*-overexpressed mutant (WT + pIJcdgA), *cdgA_gh_^AAL^*-overexpressed mutant (WT + pIJcdgA^AAL^), *cdgD_gh_*-overexpressed mutant (WT + pIJcdgD), *cdgE_gh_*-overexpressed mutant (WT + pIJcdgE), *rmdA_gh_*-overexpressed mutant (WT + pIJrmdA) and *rmdA_gh_^AAL^*-overexpressed mutant (WT + pIJrmdA^AAL^). The mean value of the moenomycin mass peak area of *S. ghanaensis* WT and *S. ghanaensis* WT + pIJ10257 was taken as 100%. Error bars represent standard deviations. Significant differences in moenomycin A (MmA) production between *S. ghanaensis* WT and each mutant strain were calculated by a two-tailed *t*-test. Asterisks represent the significance value (* *p* < 0.05, ** *p* < 0.01), whereas NS refers to not-significant differences.

In comparison to the wild-type strain (100%), *S. ghanaensis* Δ*rmdA_gh_*, *S. ghanaensis* Δ*cdgA_gh_* and *S. ghanaensis* Δ*cdgD_gh_* mutants produced on average 140%, 170%, and 136% of MmA, respectively (Figure 4A). Conversely, no changes in antibiotic levels were observed in *S. ghanaensis* Δ*cdgE_gh_* (Figure 4A). In order to exclude polar effect, complementation experiments were performed. For this purpose, pIJcdgD and pIJrmdA were used to complement Δ*cdgD_gh_* and Δ*rmdA_gh_* mutants, respectively. Instead, complementation of *S. ghanaensis* Δ*cdgA_gh_* was achieved by cloning the *cdgA_gh_* coding sequence, along with its own promoter, in the φC31-based integrative vector pSET152, yielding pSETcdgA. Null-mutants carrying an empty copy of the corresponding vector were used as control. Indeed, the introduction of a native copy of the gene could abolish the effects of *rmdA_gh_*, *cdgA_gh_* and *cdgD_gh_* knockouts on MmA production (Figure S5).

Unlike gene deletion, overexpression of *cdgA_gh_* (plasmid pIJcdgA) led to a 40% less antibiotic production with respect to the control strain, carrying an empty copy of pIJ10257 plasmid. Additionally, the introduction of pIJcdgA^AAL^ (carrying the mutated allele *cdgA_gh_^AAL^*) had no effect on MmA accumulation (Figure 4B).

Analysis of *S. ghanaensis* + pIJcdgD revealed that this strain accumulated fivefold less MmA, whereas *S. ghanaensis* + pIJcdgE showed a slight reduction (15%) of antibiotic production (Figure 4B).

Intriguingly, the overexpression of *rmdA_gh_* (plasmid pIJrmdA) led to a slight increase in MmA levels, while *S. ghanaensis* + pIJrmdA^AAL^ (carrying a mutated copy of *rmdA_gh_*) displayed around a twofold increase of MmA biosynthesis (Figure 4B).

Summarizing, our results integrate the findings previously reported [15,30] and revealed the involvement of other c-di-GMP-metabolizing enzymes in antibiotic production in *S. ghanaensis*. Particularly, we showed that deletion and overexpression of genes encoding for DGCs or PDEs can be a useful technique to vary MmA titers in *S. ghanaensis* ATCC14672.

## 4. Discussion

c-di-GMP is a well-known bacterial second messenger, reported to affect a wide range of phenotypes such as biofilm formation, sessility-to-motility transition, virulence, adhesion, and cell progression in flagellated, unicellular microorganisms [24]. Moreover, c-di-GMP has recently emerged as a crucial molecule involved in governing morphogenesis and secondary metabolite production in non-motile, multicellular *Streptomyces* species.

c-di-GMP-metabolizing enzymes have been extensively studied in the model organisms *S. coelicolor* [14,25,26,27] and *S. venezuelae* [28,29]. Orthologs of these enzymes were also identified in *S. ghanaensis* and three of them were characterized in our previous studies [15,30]. In this work, we aimed to elucidate the enzymatic activity of four remaining GGDEF/EAL proteins in *S. ghanaensis* and their role in vivo.

In line with our enzymatic in vitro assays, we showed that CdgE_gh_ is an active DGC, RmdA_gh_ is a bifunctional enzyme and CdgA_gh_ acts solely as PDE. In *S. venezuelae*, CdgE (ortholog of CdgE_gh_) was also shown to be a DGC and to undergo a substrate inhibition [29]. RmdA was first described in *S. coelicolor* exclusively as a PDE enzyme [27], whereas in *S. venezuelae* it was revealed to have double activity [29]. Similarly, we demonstrated that the GGDEF domain alone of RmdA_gh_ is able to synthesize c-di-GMP in vitro, although this molecule is immediately converted into pGpG at the presence of full-length protein. Such dominance of a PDE activity over the DGC one was also shown in the YciR protein from *E. coli* [46] and in the ortholog of RmdA_gh_ from *S. venezuelae* [29]. In agreement with previous hypothesis, we suggest that the catalytic activity of RmdA_gh_ is most likely mediated by the binding of specific signals to its PAS-PAC domain, determining thus the dominance of a function over the other. Surprisingly, we found that CdgA_gh_ is able to hydrolyse c-di-GMP through its EAL domain and has no cyclase activity. Indeed, the mutation in the highly-conservative glutamate strongly reduces its catalytic activity but does not trigger the GGDEF domain. Upon in vivo experiments, the ortholog of CdgA_gh_ in *S. coelicolor* was identified as a DGC [14], but its activity has not yet been proved in vitro. According to our *in silico* analysis, these proteins share 72% identity and both possess key residues in GGDEF and EAL domains. Nevertheless, they seem to have solely a single, opposite function in two organisms. It would be of high interest to study why these orthologs evolved different activities and how their catalysis is regulated. Both sequences possess an N-terminal PAS domain (46% identity), known to adapt to numerous ligands [47]. Due to their high plasticity, we anticipate that in *S. ghanaensis* and *S. coelicolor* CdgA activity might be controlled by two diverse environmental stimuli, which in turn differently trigger the catalytic domain and keep the other one inactive. Many examples of inactive EAL and GGDEF domains have been described previously [24]. These domains were shown to have evolved other functions, such as acting as allosteric domains [21,48] or being involved in protein–protein interactions [24]. Similarly, the inactive GGDEF domain of CdgA_gh_ might also be required to enhance the PDE activity of the cognate EAL domain or simply to stabilize the protein conformation. Finally, it cannot be excluded that the inactivity detected in our in vitro assay might be due to the lack of specific signal(s) under the tested conditions.

Morphologically, no obvious differences were detected among the four generated *S. ghanaensis* null-mutant strains. This phenomenon was also observed by Liu et al. in *S. coelicolor* [26] and suggests that c-di-GMP-metabolizing enzymes may be redundant in function. Indeed, transcriptional profile of genes encoding DGCs and PDEs in *S. venezuelae* revealed that the majority of these genes are simultaneously expressed during the entire life cycle [28]. In addition, Hull et al. showed that double mutation of *rmdA* and *rmdB* remarkably affected *S. coelicolor* phenotype in comparison to individual mutants, highlighting the overlapping functions of these two PDEs in regulating morphological development [27]. Conversely, overexpression of each studied gene severely impacted morphogenesis in *S. ghanaensis*. The arrest of development observed in both *cdgE_gh_* and *cdgD_gh_* overexpressed strains resembles the phenotypes obtained in *S. coelicolor* upon *cdgA*, *cdgB* and *cdgD* overexpression (i.e., inhibition of aerial mycelium formation) [14,25,26] and correlates with the phenotypes recently examined in *cdgB_gh_* and *cdgC_gh_* overexpressed *S. ghanaensis* strains [15,30].

Until now, BldD and the sporulation-specific σ^WhiG^ factor were shown to be two c-di-GMP sensing effectors in streptomycetes [17,18]. BldD works as a dimer and controls the expression of developmental genes [14,16,17], whereas the σ^WhiG^ factor blocks the transcription of sporulation-associated genes when bound to its anti-σ [18]. In turn, both BldD dimerization and σ^WhiG^ -anti-σ binding are mediated by c-di-GMP. Consequently, high concentrations of the second messenger—caused by the overproduction of DGC enzymes—induces the dimer formation as well as the σ^WhiG^-anti-σ interaction, resulting in the arrest of morphological progression.

On the contrary, increased hydrolysis of c-di-GMP due to an enhanced PDE activity triggers morphogenesis. For example, in *S. ghanaensis* overexpression of *rmdB_gh_* (encoding a PDE) resulted in an accelerated spore formation [15]. Similarly, in this study we demonstrated that overexpression of *rmdA_gh_* and *cdgA_gh_* remarkably led to precocious sporulation and mutations in their catalytic EAL domain could revert the obtained phenotype.

Next, we aimed to study the influence of these enzymes on secondary metabolite production. Given its high potency and broad spectrum of activity, MmA is a promising lead to combat MDR pathogens. Recently, we expanded the knowledge regarding MmA regulation, showing that variations in intracellular c-di-GMP levels strongly affect antibiotic accumulation [15,30]. Indeed, c-di-GMP is required for mediating the formation of a BldD dimer, which, in turn, blocks the transcription of *wblA* but positively regulates the expression of *adpA* [15]. WblA and AdpA were shown to have an opposite effect on MmA biosynthesis. In fact, WblA negatively regulates antibiotic production [6], whereas AdpA triggers the transcription of key structural genes in the *moe* cluster [7]. In this study, we found that deletion of *rmdA_gh_* and *cdgA_gh_* led to an increase in MmA biosynthesis, as also shown previously in the Δ*rmdB_gh_* mutant [15]. In agreement with our studies, we believe that an increment of intracellular c-di-GMP levels occurs in Δ*rmdA_gh_* and Δ*cdgA_gh_* mutants, promoting BldD dimer formation and thus its binding to the target promoters. An opposite effect was instead observed in *cdgA_gh_*-overexpressed *S. ghanaensis*.

Strikingly, overexpression of *rmdA_gh_* resulted in a slight increase of antibiotic titers. As discussed above, RmdA_gh_ is capable of both DGC and PDE activities and its overproduction might lead to a change in dominance between the two functions. Indeed, inactivation of the EAL domain by point mutation caused a more remarkable increment of MmA accumulation in comparison to Δ*rmdA_gh_* mutant. This phenotype resembled that observed in *S. ghanaensis* strains overproducing the DGCs CdgB_gh_ and CdgC_gh_, respectively [15,30], suggesting that likely an increase of c-di-GMP concentration occurred upon inactivation of RmdA_gh_ EAL domain.

In line with our *in silico* analysis, CdgD_gh_ is predicted to be a DGC, albeit we could not prove its enzymatic activity in vitro. Nevertheless, Liu et al. demonstrated that CdgD (an ortholog of CdgD_gh_) indeed functions as a cyclase and its overexpression negatively affects actinorhodin production in *S. coelicolor* [26]. Similarly, overexpression of *cdgD_gh_* greatly reduced MmA levels, whereas *S. ghanaensis* Δ*cdgD_gh_* accumulated more antibiotic. These results are somewhat puzzling considering that in *S. ghanaensis* deletion of other two active DGCs (i.e., CdgB_gh_ and CdgC_gh_) was previously shown to have an opposite effect [15,30]. In fact, in *S. ghanaensis*, a decrease of c-di-GMP levels caused a reduction in antibiotic biosynthesis. The majority of DGC and PDE enzymes contain N-terminal transmembrane and/or sensor domains associated with the catalytic ones. Exceptionally, CdgD_gh_ has only a stand-alone GGDEF domain, which lacks the RxxD inhibition site, suggesting that this enzyme might undergo an uncommon mechanism of regulation. In addition, *cdgD_gh_* is in operon with other three genes of yet-unknown functions. We speculate that *cdgD_gh_* might be a part of a local c-di-GMP-dependent micro-compartment, whose perturbation can lead to a signaling cascade involving yet-undiscovered regulators associated with antibiotic production. Numerous examples of local c-di-GMP signaling pathways were already described previously in other bacteria [49,50,51,52], and the presence of diverse c-di-GMP-metabolizing enzymes in the same organism strongly suggests that a similar mechanism exists also in streptomycetes.

Finally, among all generated *S. ghanaensis* null-mutants, Δ*cdgE_gh_* did not show any changes in MmA accumulation, but overexpression of *cdgE_gh_* slightly reduced antibiotic titers. We propose that this enzyme is not directly involved in secondary metabolism pathway and that the results obtained upon overexpression are rather due to a spillover of an excess of c-di-GMP from its local pool. Indeed, indirect effects of guanylate cyclase overexpression were previously reported [51,53]. As mentioned above, the formation of spatially organized c-di-GMP pools is highly common among bacteria. Similarly, CdgE_gh_ might be associated to a parallel and compartmentalized signaling cascade mostly implicated in morphogenesis and/or primary metabolism. In addition, CdgE_gh_ possesses an N-terminal GAF sensor domain, therefore elucidating the nature of its ligand(s) might provide further hints regarding the mechanism of action and the regulation of CdgE_gh_.

In correlation with previous studies, our work highlights the extremely intricate and complex signaling pathway involving c-di-GMP and its metabolizing enzymes. Their spatial location as well as expression profiles are key factors to fully comprehend where and how each DGC and PDE acts. Recent studies showed the existence of a negative feedback loop comprising BldD and some DGC encoding genes. For example, we demonstrated that in *S. ghanaensis* BldD binds to the promoter regions of *cdgB_gh_* and *cdgC_gh_*, repressing their transcriptions [15,30]. Similarly, promoters of *cdgA*, *cdgB*, *cdgC* (homolog of *cdgC_gh_*), and *cdgE* (homolog of *cdgE_gh_*) are under control of BldD in *S. venezuelae [29,54]*. Interestingly, multiple sequence alignment (Figure S6) showed that a putative BldD-binding site is also present in the promoter region of *cdgA_gh_* but not of *cdgE_gh_*, suggesting that diverse regulatory mechanisms evolved in different *Streptomyces* species. Indeed, several studies in *S. venezuelae* showed that other global regulators—beside BldD—control genes encoding c-di-GMP-metabolizing enzymes [55,56,57], implying that a multilayered regulatory pathway is involved in maintaining the intracellular homeostasis of this second messenger.

## 5. Conclusions

In this work, we showed that manipulation of c-di-GMP-metabolizing enzymes strongly affects both morphological development and MmA production in *S. ghanaensis*. First, we demonstrated through enzymatic in vitro assays that CdgE_gh_ is an active DGC, RmdA_gh_ is a bifunctional enzyme with a dominant PDE activity and CdgA_gh_ acts as a PDE in *S. ghanaensis*. Strikingly, the latter finding reveals new insights into the evolution of DGC and PDE enzymes among streptomycetes, since the ortholog of CdgA in *S. coelicolor* was instead proposed to have cyclase activity [14]. Next, we demonstrated that overexpression of *cdgE_gh_* and *cdgD_gh_* (encoding a predicted DGC) led to an arrest of morphological progression, whereas overproduction of PDEs RmdA_gh_ and CdgA_gh_ triggered sporogenesis. Finally, we investigated the influence of these enzymes on antibiotic production. The strongest impact was observed upon deletion of *rmdA_gh_*, *cdgA_gh_*, and *cdgD_gh_*, which led to an increase in MmA accumulation. Conversely, deletion of *cdgE_gh_* caused no changes in antibiotic titers, suggesting that this enzyme may be rather involved in some local, secondary metabolism-independent signaling pathway. Altogether, these results provide a useful approach to improving MmA levels in *S. ghanaensis* and expanding the knowledge about the function of c-di-GMP-metabolizing enzymes in governing the *Streptomyces* life cycle.

## Figures and Tables

**Table 1:** c-di-GMP-metabolizing enzymes encoded by *S. ghanaensis.*

Gene	Domain Architecture	Orthologs	References
*ssfg_00725* (*rmdA_gh_*)	PAS-PAC-GGDEF-EAL	*sco0928* (*rmdA*), *sven6830*	This work
*ssfg_02196* (*rmdB_gh_*)	6TM^1^-GGDEF-EAL	*sco5495* (*rmdB*), *sven5165*	[15]
*ssfg_04551* (*cdgA_gh_*)	PAS-GGDEF-EAL	*sco2817* (*cdgA*), *sven2604*	This work
*ssfg_03956* (*cdgB_gh_*)	GAF-PAS-PAC-GGDEF	*sco4281* (*cdgB*), *sven4034*	[15]
*ssfg_02181* (*cdgC_gh_*)	9TM-PAS-GGDEF-EAL	*sco5511*, *sven5187* (*cdgC*)	[30]
*ssfg_02707* (*cdgE_gh_*)	GAF-GGDEF	*sco4931*, *sven4602* (*cdgE*)	This work
*ssfg_02343* (*cdgD_gh_*)	GGDEF	*sco5345* (*cdgD*), *sven3999*	This work
*ssfg_02459*	5TM-HD-GYP	*sven4873*	
*ssfg_02460*	2TM-HD-GYP^2^	*sven4872*	

^1^ TM = transmembrane domain. ^2^ Due to incomplete genome sequence, the entire domain architecture cannot be predicted.

## Data Availability

The data presented in this study are available in this article and supplementary materials.

## Supplementary Materials

The following are available online at https://www.mdpi.com/2076-2607/9/2/284/s1, Figure S1: Domains architecture of CdgA_gh_, RmdA_gh_, CdgD_gh_ and CdgE_gh_. Figure S2: MS spectra of GTP, c-di-GMP and pGpG detected after the DGC and PDE in vitro assays. Figure S3: LC –MS chromatogram of CdgA_gh_^AAL^ and CdgE_gh_^AADEF^ diguanylate cyclase in vitro assays. Figure S4: Phenotype evaluation of *S. ghanaensis* wild-type (WT), *S. ghanaensis* Δ*cdgA_gh_*_(_Δ*cdgA_gh_*_)_, *S. ghanaensis* Δ*cdgE_gh_* (Δ*cdgE_gh_*), *S. ghanaensis* Δ*cdgD_gh_* (Δ*cdgD_gh_*) and *S. ghanaensis* Δ*rmdA_gh_* (Δ*rmdA_gh_*). Figure S5: Complementation experiments in *S. ghanaensis* null-mutants. Figure S6: Multiple sequence alignment of putative BldD-binding site (BldD-box) in the promoter of *cdgA_gh_* and *cdgE_gh_* and their orthologs. Table S1: Strains and plasmids used in this work. Table S2: Primers used in this work. Table S3: Buffers used in protein purification.
