# Supplementary material for: Identification and Characterization of Four c-di-GMP-Metabolizing Enzymes from Streptomyces ghanaensis ATCC14672 Involved in the Regulation of Morphogenesis and Moenomycin A Biosynthesis
Source: Microorganisms. 2021 Jan 30;9(2):284. doi: 10.3390/microorganisms9020284 (PMC7911125; doi:10.3390/microorganisms9020284)
Supplement: Supplementary file 1 [file microorganisms-09-00284-s001.pdf]

microorganisms MDPI

Supplementary information to the article

Identification and Characterization of Four c-di-GMP-Metabolizing Enzymes from *Streptomyces ghanaensis* ATCC14672 Involved in the Regulation of Morphogenesis and Moenomycin A Biosynthesis

Desirèe Nuzzo, Roman Makitrynskyy*, Olga Tsypik and Andreas Bechthold*

Pharmaceutical Biology and Biotechnology, Institute of Pharmaceutical Sciences, Albert-Ludwigs University, Freiburg 79104, Germany

* Correspondence: R.M. (email: roman.makitrynskyy@pharmazie.uni-freiburg.de), A.B. (email: andreas.bechthold@pharmazie.uni-freiburg.de)

Supplementary Table S1. Strains and plasmids used in this work.

Strains or plasmids	Description/Functions	Source
Strains		
S. ghanaensis ATCC14672	Wild-type (WT) moenomycin producer	ATCC
S. ghanaensis Δ*cdgE*$_{gh}$	WT derivative, Δ*cdgE*$_{gh}$ deletion	This work
S. ghanaensis Δ*cdgD*$_{gh}$	WT derivative, Δ*cdgD*$_{gh}$ deletion	This work
S. ghanaensis Δ*rmdA*$_{gh}$	WT derivative, Δ*rmdA*$_{gh}$ deletion	This work
S. ghanaensis Δ*cdgA*$_{gh}$	WT derivative, Δ*cdgA*$_{gh}$ deletion	This work
S. ghanaensis pIJcdgE	WT derivative, *cdgE*$_{gh}$ overexpression	This work
S. ghanaensis pIJcdgD	WT derivative, *cdgD*$_{gh}$ overexpression	This work
S. ghanaensis pIJrmdA$_{gh}$	WT derivative, *rmdA*$_{gh}$ overexpression	This work
S. ghanaensis pIJcdgA$_{gh}$	WT derivative, *cdgA*$_{gh}$ overexpression	This work
S. ghanaensis pIJrmdA^{AAL}	WT derivative, *rmdA*$_{gh}^{AAL}$ overexpression	This work
S. ghanaensis pIJcdgA^{AAL}	WT derivative, *cdgA*$_{gh}^{AAL}$ overexpression	This work
E. coli XL1Blue	Host strain for DNA cloning	Agilent
E. coli ET12567 (pUZ8002)	Host for *E. coli*-streptomycetes conjugation	[1]
E. coli BW25113	Host for REDIRECT technology with helper plasmid pIJ790	[2]
E. coli BL21 Star (DE3)	Host for protein production	Thermo Fisher Scientific
E. coli BL21 Star (DE3)/pLysS	Host for protein production	Thermo Fisher Scientific
Plasmids		
pBluesctiptIIKS+	Cloning vector, Ap^R	Addgene
pKGLP2	Suicide vector carrying *gusA*, Hyg^R	[3]
pSET152	φC31-based integrative vector, Am^R	[4]
pUWLCre	Vector carrying *cre* under *ermEp*, Tsr^R	[5]
pIJ10257	φBT1-based integrative vector carrying *ermEp*, Hyg^R	[6]
pLERECJ	Vector carrying apramycin resistance cassette with *loxP*-sites for gene replacement	Prof. Luzhetskyy, Saarland University
pET28a	Vector for protein production, Km^R	Novagen
pET32a	Vector for protein production, Ap^R	Novagen
pBluecdgE	pBluescriptIIKS+ carrying *cdgE*$_{gh}$	This work
pBluecdgD	pBluescriptIIKS+ carrying *cdgD*$_{gh}$	This work
pBluermdA	pBluescriptIIKS+ carrying *rmdA*$_{gh}$	This work
pBluecdgA	pBluescriptIIKS+ carrying *cdgA*$_{gh}$	This work
pBluecdgE::aac(3)IV	pBlue02707, Δ*cdgE*$_{gh}$ deletion	This work
pBluecdgD::aac(3)IV	pBlue02343, Δ*cdgD*$_{gh}$ deletion	This work

pBluermdA::aac(3)IV	pBluermdA, $\Delta rmdA_{gh}$ deletion	This work
pBluecdgA::aac(3)IV	pBluecdgA, $\Delta cdgA_{gh}$ deletion	This work
pSETcdgA	pSET152 carrying $cdgA_{gh}$ along with its promoter	This work
pKGcdgE::aac(3)IV	pKGLP2 carrying *cdgE::aac(3)IV*	This work
pKGcdgD::aac(3)IV	pKGLP2 carrying *cdgD::aac(3)IV*	This work
pKGrmdA::aac(3)IV	pKGLP2 carrying *rmdA::aac(3)IV*	This work
pKGcdgA::aac(3)IV	pKGLP2 carrying *cdgA::aac(3)IV*	This work
pIJcdgE	pIJ10257 carrying $ermEp\text{-}cdgE_{gh}$ fusion	This work
pIJcdgD	pIJ10257 carrying $ermEp\text{-}cdgD_{gh}$ fusion	This work
pIJrmdA	pIJ10257 carrying $ermEp\text{-}rmdA_{gh}$ fusion	This work
pIJcdgA	pIJ10257 carrying $ermEp\text{-}cdgA_{gh}$ fusion	This work
pIJrmdA^{AAL}	pIJ10257 carrying a mutated version of $rmdA_{gh}$	This work
pIJcdgA^{AAL}	pIJ10257 carrying a mutated version of $cdgA_{gh}$	This work
pETcdgE	pET28b carrying His-tagged CdgE$_{gh}$	This work
pETcdgE^{AADEF}	pET28b carrying a mutated version of CdgE$_{gh}$	This work
pETrmdA	pET28b carrying His-tagged RmdA$_{gh}$	This work
pETcdgA	pET28b carrying His-tagged CdgA$_{gh}$	This work
pETrmdA^{GGDEF}	pET28b carrying solely the His-tagged GGDEF domain of RmdA$_{gh}$	This work
pETrmdA^{AAL}	pET28b carrying a mutated version of His-tagged RmdA$_{gh}$	This work
pETcdgA^{AAL}	pET28b carrying a mutated version of His-tagged CdgA$_{gh}$	This work
pET32acdgA^{AAL}	pET32a carrying a mutated version of Trx-His-tagged CdgA$_{gh}$	This work

Supplementary Table S2. Primers used in this work.

**Primers**	**Sequence**
02707_del_for	AAATCTAGAAACGACGAGACGATGCCG
02707_del_rev	AAATCTAGATACGGGTGGAGGCGCTCG
02707_kn_for	AATGTACCCGTTCGCCCGGAATCTCCCTAGCCTGGAGGGAT GGATATCTCTAGATACCG
02707_kn_rev	GCGCGTCACACCCGCCGGAGCCGCGGCGGGGCGTGCGGTC AAACAAAAGCTGGAGCTC
02343_del_for	AAATCTAGAAGTCTGTAGGTGCATCGAGC
02343_del_rev	AAAGAATTCAAGAGCACCTGGCACTCG
02343_kn_for	GCTCCTTGCCGAACGCGCCGCCGGCGCGGGGGTGAGCGCA TGAGCGATATCTCTAGATACCG
02343_kn_rev	GTCCGGGGCCAGGTGCCACCGGAACACCGGCAGCGCACTC ATGCAACAAAAGCTGGAGCTC
cdgA_del_for	AAATCTAGAGTGGTGATCTTCACCGTCCAC
cdgA_del_rev	AAAGAATTCCTGCAGGAGATCGAGGTGC
cdgA_kn_for	CTGCACGGACAGCGATCGAGTGCACTGCGGGAGCGAGAGG TGGATATCTCTAGATACCG
cdgA_kn_rev	CTACGGGTTGCCGGCCCCCGCCTGAACCCCGGACGCGCCCA GAACAAAAGCTGGAGCTC
rmdA_del_for	AAATCTAGAGAGTTGACGATGAATACCTCCT
rmdA_del_rev	AAAGAATTCAAGTCATGTTCAGCTCACCA
rmdA_kn_for	CGCCTTCTCCGGCTTCGCGGGCGTGGGGCGTACGGCGTGAG CGATATCTCTAGATACCG
rmdA_kn_rev	CACCGATCGTCCGGCCGGTCCGCGACCGGTTTGCGCCTCAA ACAAAAGCTGGAGCTC
cdgA_compl_for	AAATCTAGAGACGGTTCACGGGACACCG
cdgA_compl_rev	AAAGAATTCGTGCGGCTGCGCTCCTAC
02707_exp_for	AAAAAACATATGGGTGAGGACAGCCGGCT

02707_exp_rev	AAACTCGAGTCACGGCGAGTGCCGCCCCC
02343_exp_for	AAAAAACATATGAGCACCACGGCACTTGC
02343_exp_rev	AAACTCGAGTCATGCCGCGCCGCGGCGGC
rmdA_exp_for	AAAAAACATATGAGCGTGGAACCGGACGGG
rmdA_exp_rev	AAAAAGCTTTCACCCCGACGCGTCCAC
cdgA_exp_for	AAAAAACATATGGTGAGCGGAACGTCCGA
cdgA_exp_rev	AAACTCGAGCTACGGGTTGCCGGCCCCCG
rmdA_GGDEF_rev	AAAAAGCTTTCAGTCGGCGAGCTCGAACCG
cdgA_AAL_for	GCGGCCCTGGTCCGCTGGAACC
cdgA_AAL_rev	CACGCCGTGCACCCGG
rmdA_AAL_for	GCGGCGCTGGTGCGCTGGC
rmdA_AAL_rev	GGCGCCGCGGACGCTC
02707_AADEF_for	GCCGCCGACGAGTTCTGCCTGCTGTCC
02707_AADEF_rev	GAGCCGCGCCGCCAGG

Supplementary Table S3. Buffers used in protein purification.

Protein	Equilibration buffer	Storage buffer
$CdgE_{gh}$<br>$CdgE_{gh}^{AADEF}$	50 mM TrisHCl (pH 7.0)<br>0.5 M NaCl<br>5 mM Imidazole	50 mM TrisHCl (pH 7.0)<br>0.5 M NaCl<br>1 mM $MgCl_2$<br>1 mM DTT<br>10% Glycerol
$RmdA_{gh}$<br>$CdgA_{gh}$<br>$RmdA^{GGDEF}$<br>$CdgA_{gh}^{AAL}$	50 mM TrisHCl (pH 7.5)<br>0.25 M NaCl<br>1 mM $MgCl_2$<br>5 mM β-mercaptoethanol<br>5% Glycerol<br>10 mM Imidazole	50 mM TrisHCl (pH 7.5)<br>0.25 M NaCl<br>1 mM $MgCl_2$<br>1 mM DTT<br>5% Glycerol

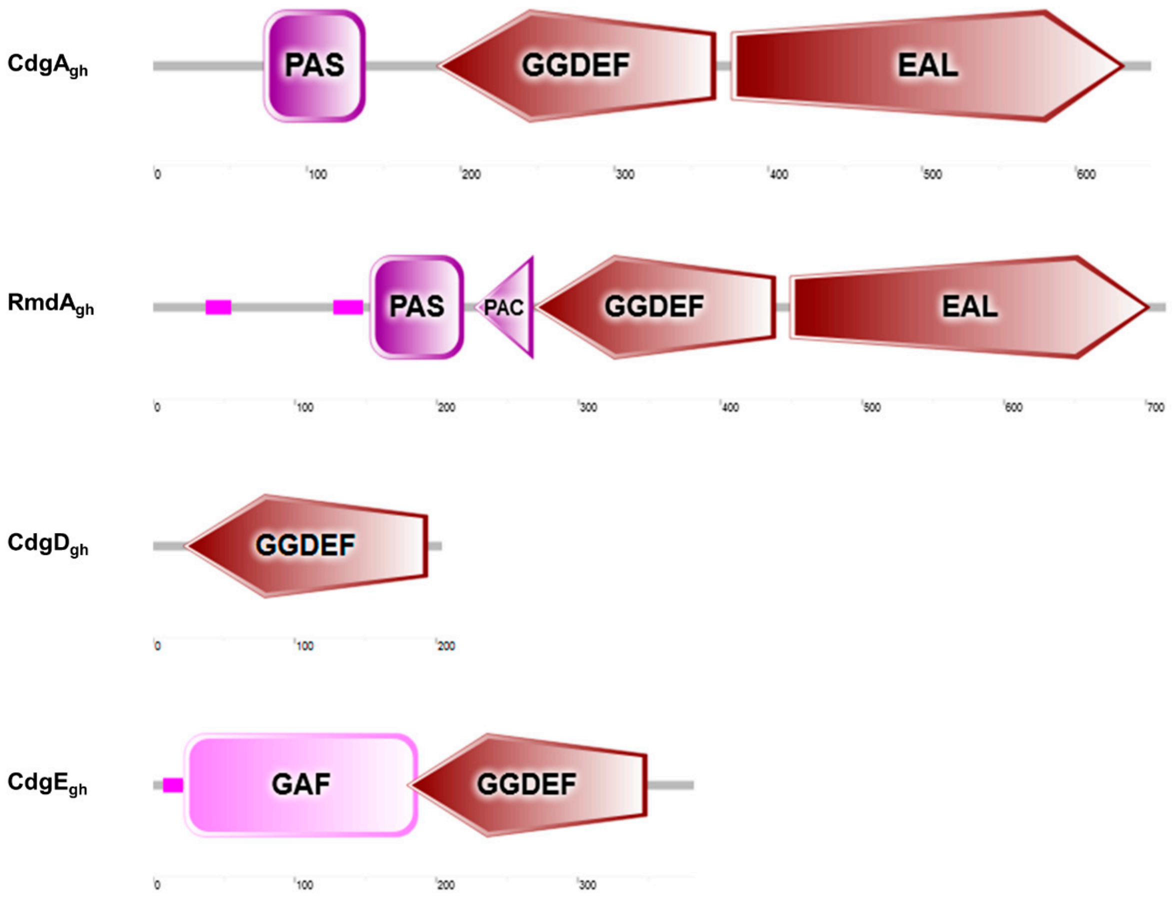

Figure S1. Domains architecture of CdgA$_{gh}$, RmdA$_{gh}$, CdgD$_{gh}$ and CdgE$_{gh}$. Domains composition was predicted by the SMART database (https://smart.embl.de).

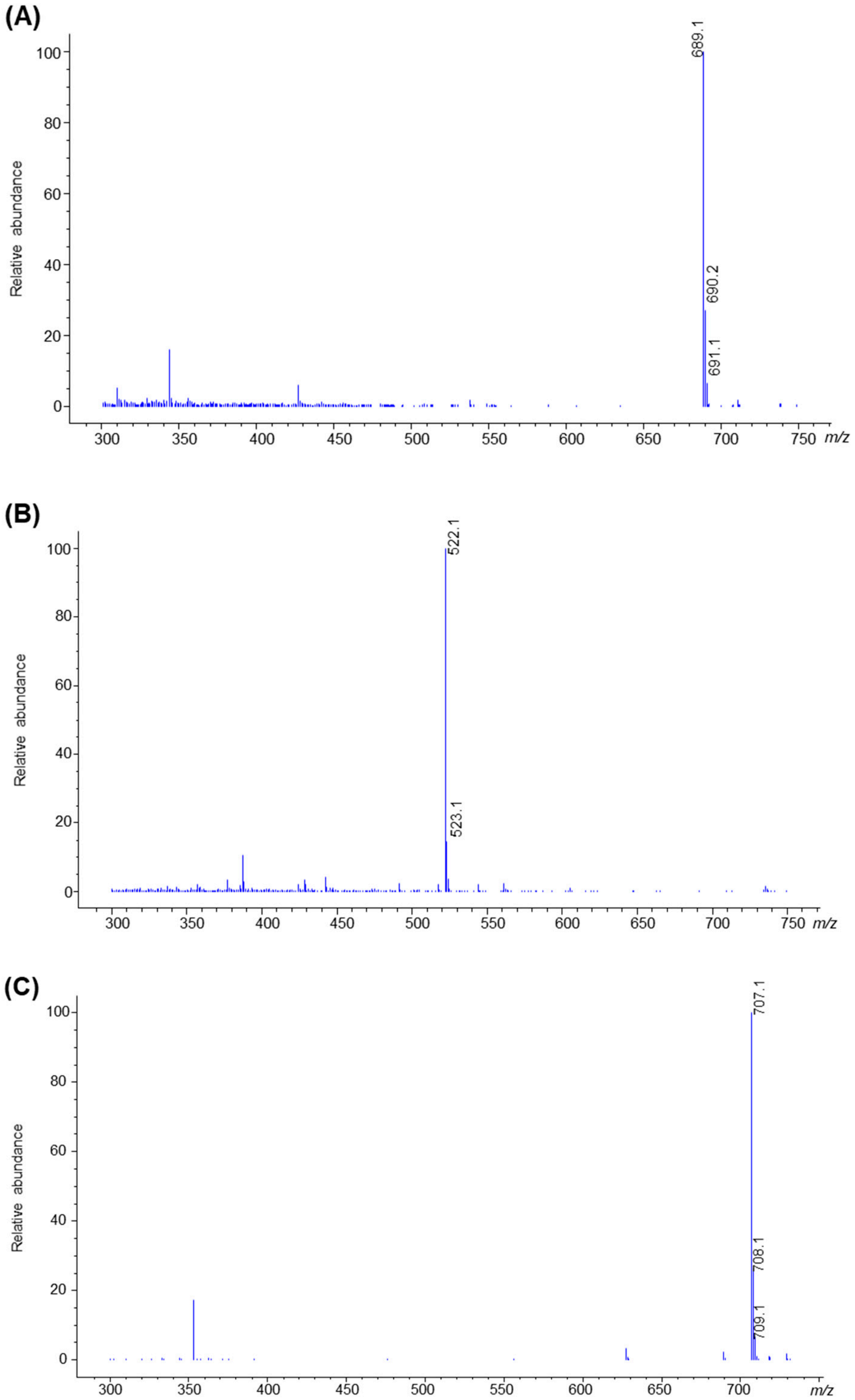

Figure 2. MS spectra of GTP, c-di-GMP and pGpG detected after the DGC and PDE *in vitro* assays. **A**) [M-H]- ion of GTP (*m*/*z* 522.1). **B**) [M-H]- ion of c-di-GMP (*m*/*z* 689.1). **C**) [M-H]- ion of pGpG (*m*/*z* 707.1).

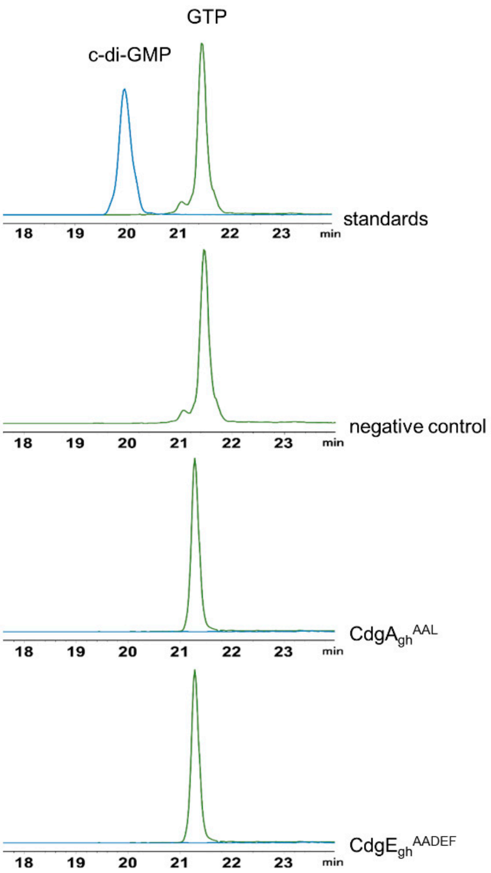

Figure 3. LC-MS chromatograms of CdgA$_{gh}^{AAL}$ and CdgE$_{gh}^{AADEF}$ diguanylate cyclase *in vitro* assays. No conversion of GTP into c-di-GMP ([M-H]$^-$ ion of c-di-GMP (*m*/*z*= 689.1)) was observed in both reaction mixtures containing CdgA$_{gh}^{AAL}$ and CdgE$_{gh}^{AADEF}$, respectively.

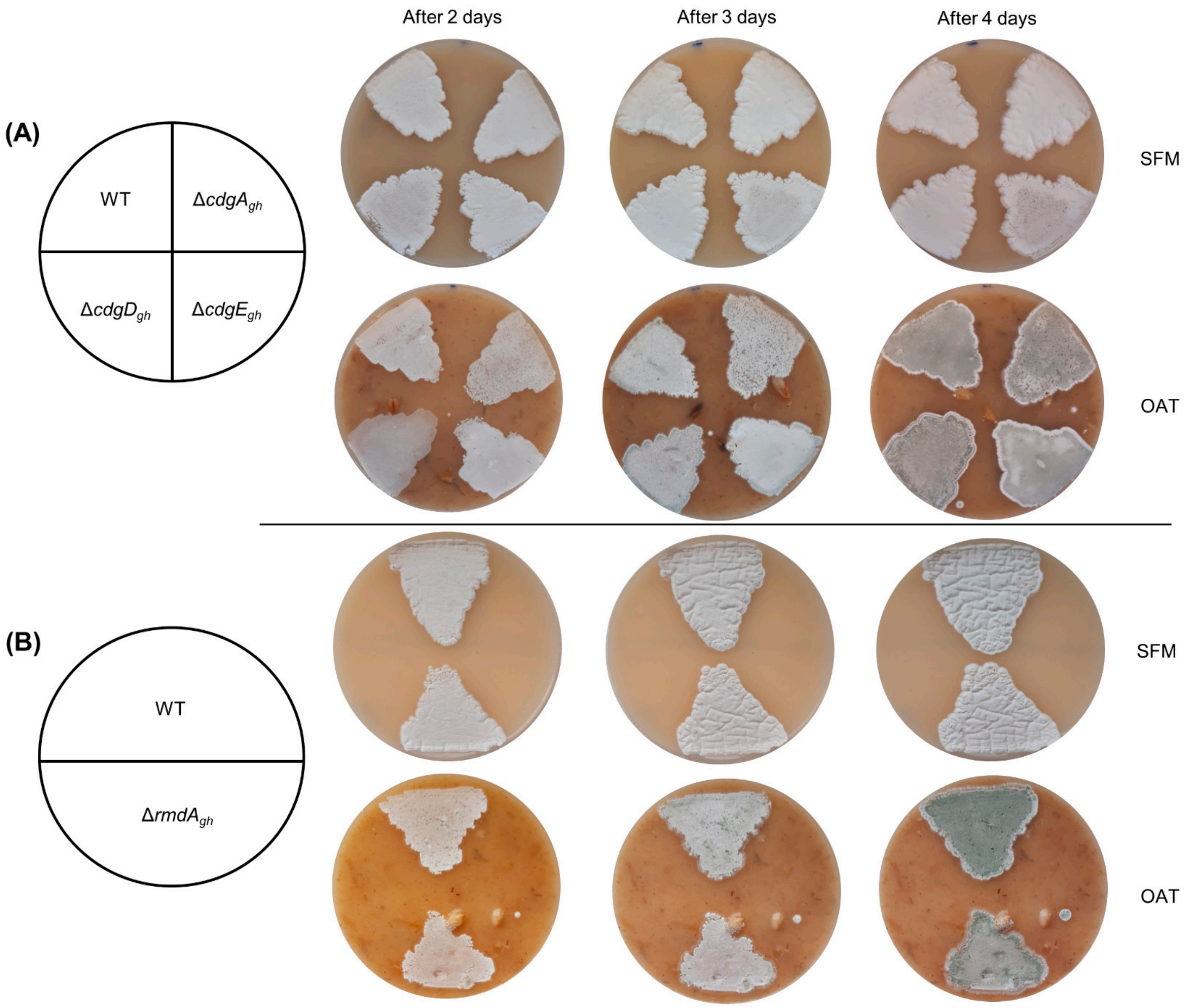

Figure 4. Phenotype evaluation of *S. ghanaensis* wild-type (WT), *S. ghanaensis* Δ*cdgA*$_{gh}$ (Δ*cdgA*$_{gh}$), *S. ghanaensis* Δ*cdgE*$_{gh}$ (Δ*cdgE*$_{gh}$), *S. ghanaensis* Δ*cdgD*$_{gh}$ (Δ*cdgD*$_{gh}$) and *S. ghanaensis* Δ*rmdA*$_{gh}$ (Δ*rmdA*$_{gh}$). In comparison to the control strain (WT), no obvious differences in morphological development were observed upon inoculation of *S. ghanaensis* Δ*cdgA*$_{gh}$, *S. ghanaensis* Δ*cdgE*$_{gh}$, *S. ghanaensis* Δ*cdgD*$_{gh}$ (**A**) and *S. ghanaensis* Δ*rmdA*$_{gh}$ (**B**) in SFM and oatmeal (OAT) agar after four days of growth.

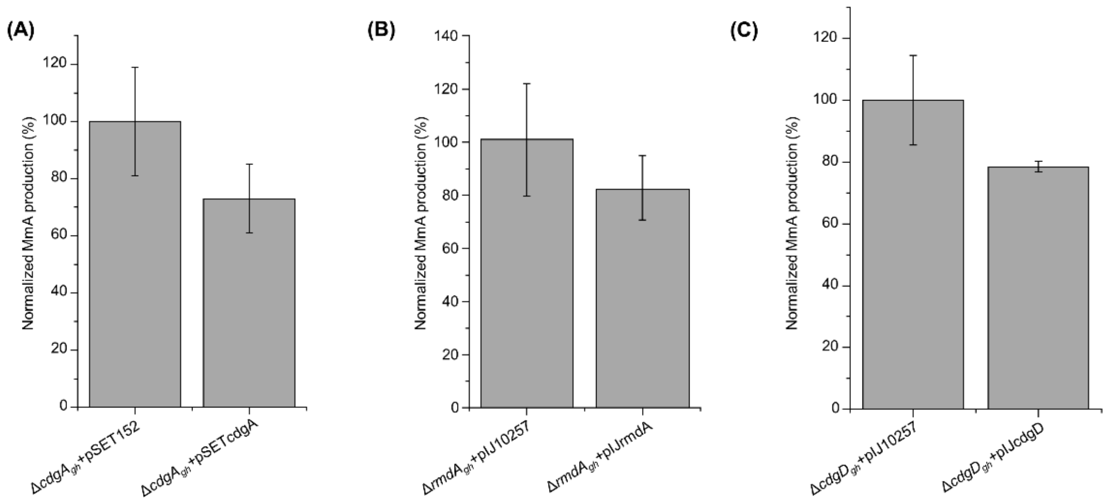

Figure 5. Complementation experiments in *S. ghanaensis* null-mutants. **A**) *S. ghanaensis* Δ*cdgA*$_{gh}$ carrying an empty copy of pSET152 (Δ*cdgA*$_{gh}$+pSET152) and complemented strain (Δ*cdgA*$_{gh}$+pSETcdgA). **B**) *S. ghanaensis* Δ*rmdA*$_{gh}$ carrying an empty copy of pIJ10257 (Δ*rmdA*$_{gh}$+pIJ10257) and complemented strain (Δ*rmdA*$_{gh}$+pIJrmdA). **C**) *S. ghanaensis* Δ*cdgD*$_{gh}$ carrying an empty copy of pIJ10257 (Δ*cdgD*$_{gh}$+pIJ10257) and complemented strain (Δ*cdgD*$_{gh}$+pIJcdgD). The mean value of moenomycin mass peak area of each *S. ghanaensis* null-mutant carrying the empty vector was taken as 100%. Error bars represent standard deviations.

BldD-box

nTnACnC(A/T)GnGTnAn

(A)

```
cdgAp vnz      5′-CGGAAAGTCACATTCGGTCACGTCGT-3′   -215
               3′-GCCTTTCAGTGTAAGCCAGTGCAGCA-5′

cdgAp coe      5′-AAGACACTCACGGAACGTCACAACTG-3′   -66
               3′-TTCTGTGAGTGCCTTGCAGTGTTGAC-5′

cdgAp gh       5′-GCACCACGCACGGAACGTCATAACTG-3′   -171
               3′-CGTGGTGCGTGCCTTGCAGTATTGAC-5′
                        *   ***      ****
```

(B)

```
cdgEp vnz      5′-CTAAATGTGACTTACGGTGACGT----------CTC-3′   -59
               3′-GATTTACACTGAATGCCACTGCA----------GCG-5′

sco4931p coe   5′-ACACGACCCTACGAGCCGAAGGCAATCGCCCGGTTC-3′
               3′-TGTGCTGGGATGCTCGGCTTCCGTTAGCGGGCCAAG-5′

cdgEp gh       5′-TCCCGGAACCGCCCCCGTCGCGGGCGGTCCCCGTTC-3′
               3′-AGGGCCTTGGCGGGGGCAGCGCCCGCCAGGGGCAAC-5′
```

Figure 6. Multiple sequence alignment of putative BldD-binding site (BldD-box) in the promoter of $cdgA_{gh}$ and $cdgE_{gh}$ and their orthologs. **A**) Alignment of BldD-boxes (in bold) identified in the promoter region of *cdgA* in *S. venezuelae* ($cdgAp_{vnz}$) [7,8] and *S. coelicolor* ($cdgAp_{coe}$) [9] shows a putative BldD-box also present in the promoter of $cgdA_{gh}$ ($cdgAp_{gh}$). Asterisks indicate identical nucleotides. **B**) A BldD-box was identified in the promoter of *cdgE* in *S. venezuelae* ($cdgEp_{vnz}$) [7,8] but not in the promoter regions of its orthologs from *S. coelicolor* ($cdgEp_{coe}$) and *S. ghanaensis* ($cdgEp_{gh}$). BldD-box was determined by den Hengst et al. (2010) [9] and the numbers represent the distance from the putative start codon of the corresponding downstream gene. .

References

1. Kieser, T. Practical streptomyces genetics; John Innes Foundation: Norwich, 2000, ISBN 0708406238.
2. Gust, B.; Chandra, G.; Jakimowicz, D.; Yuqing, T.; Bruton, C.J.; Chater, K.F. λ Red-Mediated Genetic Manipulation of Antibiotic-Producing Streptomyces. In Advances in applied microbiology; Laskin, A.I., Bennett, J.W., Gadd, G.M., Eds.; Elsevier: Estados Unidos, 2004; pp 107–128, ISBN 9780120026562.
3. Myronovskyi, M.; Welle, E.; Fedorenko, V.; Luzhetskyy, A. β-Glucuronidase as a Sensitive and Versatile Reporter in Actinomycetes. Appl. Environ. Microbiol. **2011**, *77*, 5370–5383, doi:10.1128/AEM.00434-11.
4. Bierman, M.; Logan, R.; O'Brien, K.; Seno, E.T.; Nagaraja Rao, R.; Schoner, B.E. Plasmid cloning vectors for the conjugal transfer of DNA from Escherichia coli to Streptomyces spp. Gene **1992**, *116*, 43–49, doi:10.1016/0378-1119(92)90627-2.
5. Fedoryshyn, M.; Welle, E.; Bechthold, A.; Luzhetskyy, A. Functional expression of the Cre recombinase in actinomycetes. Appl. Microbiol. Biotechnol. **2008**, *78*, 1065–1070, doi:10.1007/s00253-008-1382-9.
6. Tschowri, N.; Schumacher, M.A.; Schlimpert, S.; Chinnam, N.B.; Findlay, K.C.; Brennan, R.G.; Buttner, M.J. Tetrameric c-di-GMP mediates effective transcription factor dimerization to control Streptomyces development. Cell **2014**, *158*, 1136–1147, doi:10.1016/j.cell.2014.07.022.
7. Haist, J.; Neumann, S.A.; Al-Bassam, M.M.; Lindenberg, S.; Elliot, M.A.; Tschowri, N. Specialized and shared functions of diguanylate cyclases and phosphodiesterases in Streptomyces development. Mol. Microbiol. 2020, doi:10.1111/mmi.14581.

8. 6. Hong, H.-J.; Hutchings, M.I.; Hill, L.M.; Buttner, M.J. The role of the novel Fem protein VanK in vancomycin resistance in Streptomyces coelicolor. J. Biol. Chem. **2005**, *280*, 13055–13061, doi:10.1074/jbc.M413801200.
9. Den Hengst, C.D.; Tran, N.T.; Bibb, M.J.; Chandra, G.; Leskiw, B.K.; Buttner, M.J. Genes essential for morphological development and antibiotic production in Streptomyces coelicolor are targets of BldD during vegetative growth. Mol. Microbiol. **2010**, *78*, 361–379, doi:10.1111/j.1365-2958.2010.07338.x.
